# Significance of filamin A in mTORC2 function in glioblastoma

**DOI:** 10.1186/s12943-015-0396-z

**Published:** 2015-07-02

**Authors:** Naphat Chantaravisoot, Piriya Wongkongkathep, Joseph A. Loo, Paul S. Mischel, Fuyuhiko Tamanoi

**Affiliations:** Department of Microbiology, Immunology & Molecular Genetics, University of California, Los Angeles, CA 90095 USA; Department of Chemistry and Biochemistry, University of California, Los Angeles, CA 90095 USA; Department of Biological Chemistry, University of California, Los Angeles, CA 90095 USA; Jonsson Comprehensive Cancer Center, University of California, Los Angeles, CA 90095 USA; UCLA/DOE Institute of Genomics and Proteomics, University of California, Los Angeles, CA 90095 USA; Ludwig Institute for Cancer research, University of California, San Diego, CA 92093 USA

**Keywords:** Glioblastoma Multiforme, mTORC2, RICTOR, Filamin A, PP242, Actin cytoskeleton

## Abstract

**Background:**

Glioblastoma multiforme (GBM) is one of the most highly metastatic cancers. GBM has been associated with a high level of the mechanistic target of rapamycin complex 2 (mTORC2) activity. We aimed to observe roles of mTORC2 in GBM cells especially on actin cytoskeleton reorganization, cell migration and invasion, and further determine new important players involved in the regulation of these cellular processes.

**Methods:**

To further investigate the significance of mTORC2 in GBM, we treated GBM cells with PP242, an ATP-competitive inhibitor of mTOR, and used RICTOR siRNA to knock down mTORC2 activity. Effects on actin cytoskeleton, focal adhesion, migration, and invasion of GBM cells were examined. To gain insight into molecular basis of the mTORC2 effects on cellular cytoskeletal arrangement and motility/invasion, we affinity purified mTORC2 from GBM cells and identified proteins of interest by mass spectrometry. Characterization of the protein of interest was performed.

**Results:**

In addition to the inhibition of mTORC2 activity, we demonstrated significant alteration of actin distribution as revealed by the use of phalloidin staining. Furthermore, vinculin staining was altered which suggests changes in focal adhesion. Inhibition of cell migration and invasion was observed with PP242. Two major proteins that are associated with this mTORC2 multiprotein complex were found. Mass spectrometry identified one of them as Filamin A (FLNA). Association of FLNA with RICTOR but not mTOR was demonstrated. Moreover, *in vitro,* purified mTORC2 can phosphorylate FLNA likewise its known substrate, AKT. In GBM cells, colocalization of FLNA with RICTOR was observed, and the overall amounts of FLNA protein as well as phosphorylated FLNA are high. Upon treatments of RICTOR siRNA or PP242, phosphorylated FLNA levels at the regulatory residue (Ser2152) decreased. This treatment also disrupted colocalization of Actin filaments and FLNA.

**Conclusions:**

Our results support FLNA as a new downstream effector of mTORC2 controlling GBM cell motility. This new mTORC2-FLNA signaling pathway plays important roles in motility and invasion of glioblastoma cells.

**Electronic supplementary material:**

The online version of this article (doi:10.1186/s12943-015-0396-z) contains supplementary material, which is available to authorized users.

## Background

Glioblastoma multiforme (GBM) is classified as a grade IV type for malignant gliomas according to the World Health Organization (WHO) system [1]. It is one of the most lethal tumors based on survival and recurrence rates after diagnosis and resistant to both chemo- and radiotherapeutics. In the past, most cases after initial treatments of GBM such as surgery, radio- and chemotherapy are occasionally followed by recurring tumors which eventually progress to death [2]. The epidermal growth factor receptor (EGFR) is one of the key contributors that contribute to cancer hallmarks in GBM [3]. EGFR activating mutations are commonly observed in GBM patients and the EGFR variant III is the most common type of mutation conferring constitutive signals to many pathways downstream, mainly PI3K-AKT-mTOR, and MAPK [4–8].

In brain cells, the mechanistic target of rapamycin complex 2 (mTORC2) has been implicated as one of the main regulators. Abnormality of mTORC2 signaling pathway is linked to malignant glioma and has been broadly studied [9–12]. Several mutations, ablations, deregulated signaling pathways including mTORC2 are found to be key characteristics of malignant glioma [13]. Also, mTORC2 is believed to maintain neuron morphology and synapse function which is lacking or deregulated in brain diseases [14]. Furthermore, hyperactivated mTORC2 with RICTOR overexpression was evidenced in various types of brain cancers. Masri *et al.* [15] showed that elevated mTORC2 activity promotes tumorigenesis, tumor growth and proliferation. Levels of RICTOR protein and mTORC2 activity can be related to specific stages of cancer invasiveness [15, 16]. The mTORC2 has been linked to metabolic reprogramming in GBM including glycolytic metabolism, glutaminolysis, lipogenesis, and nucleotide and ROS metabolism [17]. These activations of several pathways by mTORC2 are believed to cause resistance to signaling inhibitors [18]. Recently, TORC2 in yeast and mTORC2 have also been reported to be new important players in DNA damage control and genome stability. These processes are essential for survival of cancer cells during stress-mediated DNA damage and also for cancer development [19–22].

The mTOR complex 2 (mTORC2) consists of the four main components which are mTOR, RICTOR, mSIN1 and mLST8 (GβL), and including other closely associated proteins such as PROTOR1/2, DEPTOR, TTI1 and TEL2 [23]. mTOR is a serine-threonine protein kinase that belongs to the phosphoinositide-3-kinase-related kinase family [24]. The complex is stimulated by growth factors critical for AKT activation resulting in the phosphorylation of AKT residue Ser473 [25]. Activated AKT further signals downstream to contribute to events such as cell survival, apoptosis, lipid and glucose metabolisms, and via mTORC1 to promote cell proliferation, and protein synthesis [26, 27]. On the other hand, mTORC2 has also been shown to control actin cytoskeleton reorganization independent of AKT [28, 29]. One mechanism is by phosphorylating the hydrophobic motif of SGK1 and several isoforms of PKCs [13, 30, 31] that are important for transcription regulation, actin cytoskeleton reorganization [17]. Moreover, mTORC2-mediating actin polymerization is required for the regulation of both long-term memory formation and long-term synaptic plasticity in mice [32]. Depletion of mTOR or RICTOR but not RAPTOR was recently demonstrated to impair migration, invasion, and stress fiber formation of highly migratory *Tsc2*^*-/-*^ mouse embryonic fibroblast cells [33]. However, mechanisms that mTORC2 use to regulate morphology and motility of cells are still poorly understood.

To gain further insight into the function of mTORC2, we first examined effects of inhibiting mTORC2 by using the mTOR kinase inhibitor PP242 [34]. This revealed alteration of cellular cytoskeleton as well as focal adhesions and inhibition of motility and invasion. Affinity purification and characterization of mTORC2 from GBM cells were performed and these led to the finding that Filamin A (FLNA) is associated with mTORC2 through its binding to RICTOR. In fact, FLNA is a substrate of mTORC2. RICTOR knockdown in GBM cells as well as PP242 treatment resulted in the inhibition of phosphorylation of FLNA. The PP242 treatment contributes to the disruption of colocalization of FLNA and actin. Taken together, these results suggest that mTORC2 affects FLNA and results in changes in motility and invasion in GBM.

## Results

### mTOR inhibitor PP242 inhibits mTORC2 activity and affects actin cytoskeleton and focal adhesion

**Fig. 1:**
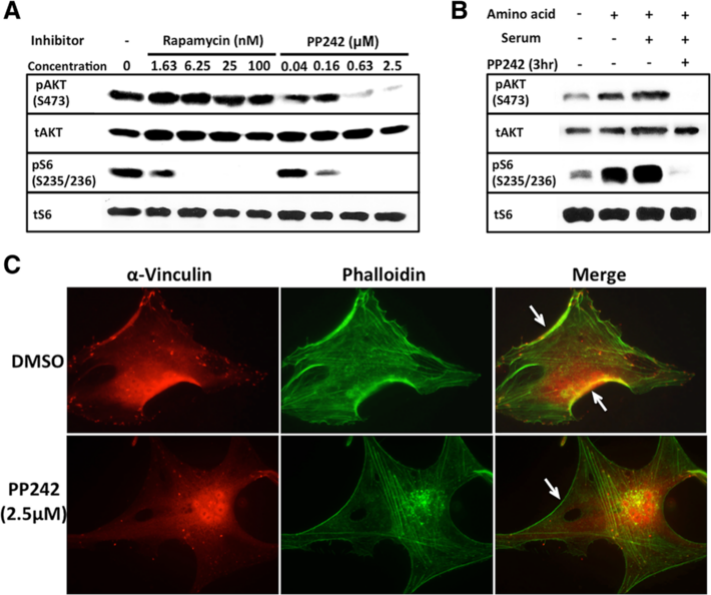
PP242 inhibits mTORC2 and exerts significant effects on actin cytoskeleton and focal adhesion of U87vIII cells. **a** PP242 inhibits mTORC1 and mTORC2 activities effectively while rapamycin only inhibits mTORC1 activity. Cells were treated with inhibitors for 24 h and phosphorylation of AKT and S6 was examined as described in Methods. **b** U87vIII cells have constitutively active mTORC1 and mTORC2 activities in starved condition. Stimulation by amino acids and serum fully activate mTOR kinase activity. A 3-h treatment of 2.5 μM PP242 ablates phosphorylation of AKT and S6. **c** Actin cytoskeleton and focal adhesion of U87vIII cells treated with PP242 for 24 h were examined using immunofluorescence staining by Phalloidin (actin) and anti-vinculin (focal adhesion) (Exposure time: actin 254 ms; vinculin 251 ms). Effects of PP242 on these processes were examined by treating cells with PP242. Effects on actin cytoskeleton rearrangement are clearly visible on the thickness of actin layers on surrounding cell membrane (arrows)

A GBM cell line U87vIII was used. This cell line is believed to be a good model for GBM that expresses EGF receptor variant III (EGFR vIII) [5, 35]. Treatment of these cells with PP242, an ATP-competitive inhibitor of mTOR kinase, causes significant decrease of mTORC2 and mTORC1 activities, as detected by the phosphorylation of AKT at Ser473 and S6 at Ser235/236, respectively (Fig. 1a). On the other hand, mTORC2 is relatively insensitive to rapamycin. These results were further confirmed using starvation condition. Under starvation, U87vIII cells have constitutively active mTOR pathway which can be fully enhanced upon nutrients and amino acids stimulation. The highly phosphorylated downstream effectors of both mTOR complexes were eliminated after a 3-h treatment of PP242 (Fig. 1b). The levels of total AKT and S6 remain the same. PP242 at the concentrations we used did not inhibit MEK and PAK1 activities, as detected by pERK (Thr202/Tyr204) and pCRKII (Ser41), respectively (Additional file 1: Figure S1A).

PP242 has significant effects on actin cytoskeleton and focal adhesion. As shown in Fig. 1c, phalloidin staining of GBM cells revealed the significant alteration of actin cytoskeleton. While actin cytosekeleton was detected predominantly on cell periphery without PP242 treatment, intracellular actin became predominant after the PP242 treatment. Besides, the thickness of membrane-bound actin filaments became significantly thinner and disordered. Staining with antibody against vinculin, a major component of focal adhesion, showed that cells lost their focal adhesions along the membrane after the PP242 treatment. We also found that anti-vinculin staining overlaps with phalloidin staining at the membrane. These results demonstrate that the inhibition of mTORC2 is associated with significant changes in cellular actin cytoskeleton and focal adhesion.

### mTOR inhibitor PP242 blocks cell migration and invasion capability of glioblastoma cells

**Fig. 2:**
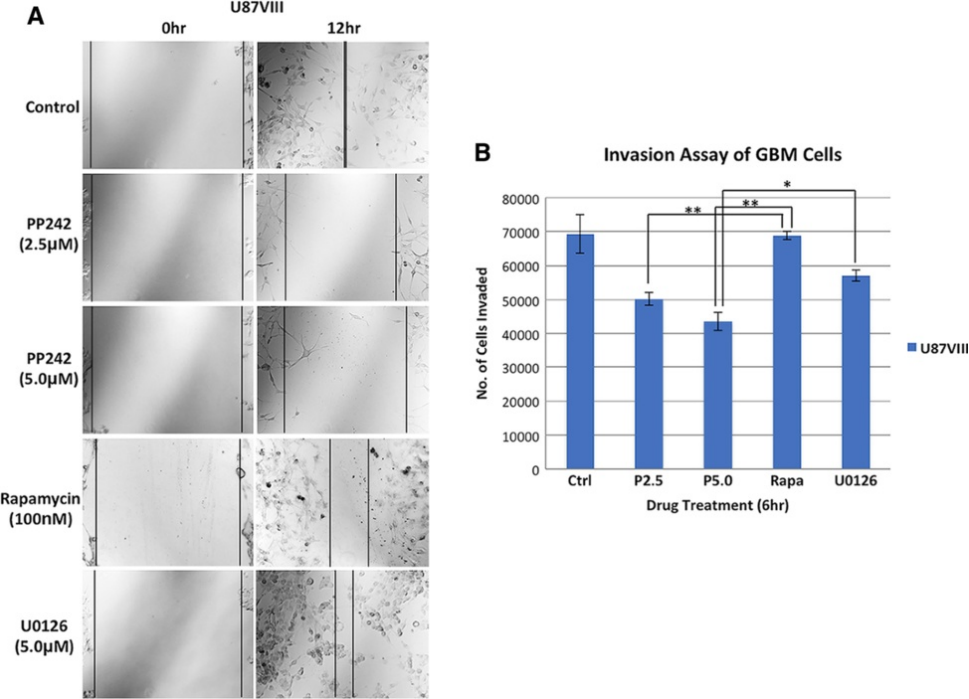
mTOR inhibition affects cell migration and invasion. **a** Wound-healing migration assay of U87vIII cells grown in a single layer. Space between a scratch is approximately 1 mm wide. Pictures were taken at 12 h after the scratch was made for each sample. Migration of cells treated with PP242, rapamycin, or U0126 was examined. **b** Numbers of invaded U87vIII cells through modified Boyden chambers were determined as described in Methods. Effects of PP242 at 2.5 and 5.0 μM, rapamycin (100 nM) or U0126 (10.0 μM) are shown. Cells were incubated with the inhibitor for 6 h. Invaded cells attaching to the chamber membrane were stained and the number of cells for each sample group was determined by measuring the absorbance values of crystal violet-stained cells at 590 nm. (P2.5 = PP242 2.5 μM, P5.0 = PP242 5.0 μM, RAPA = rapamycin 100 nM, U10 = U0126 10.0 μM) Data are the mean ± SD (*n* = 3). *P < 0.05; **P < 0.005, *P-*values from two-tailed student *t-*test (unpaired comparison)

We found that PP242 has a major effect on motility of GBM cells. Fig. 2a shows the result of scratch wound healing assays to investigate effects of mTORC2 inhibition on cell migration. Prior to the assays, we confirmed that PP242 did not affect cell density after a 24-h treatment (Additional file 2: Figure S2A). While U87vIII cells closed the gap caused by scratch wounding after 12 h (control), PP242 treatment significantly inhibited gap closing in a dose-dependent manner, suggesting that cell movement was inhibited (compare the distance that cells moved at 12 h after the scratch was made). This result is in contrast to the treatment with rapamycin that exhibited much weaker inhibition of motility. Since rapamycin predominantly affects mTORC1 under the condition used, inhibition of mTORC2 appears to contribute to the PP242 effects. Proliferation inhibition is less than 30 % under this condition (data not shown). Also, treatment with MEK inhibitor U0126 did not exhibit significant inhibition of motility in this scratch wound assay. These results suggest that mTORC2 plays a major role in GBM cell migration.

The effects of PP242 on the ability of GBM cells to invade through extracellular matrix were examined using modified transwell Boyden chambers filled with basement membrane extract. U87vIII cells were treated with PP242, U0126 or rapamycin for 6 h followed by the incubation in no-serum media without drugs. Within this time frame, proliferation inhibition is minimal. Invaded cells on the membrane were detected by fixing and staining with crystal violet (Additional file 2: Figure S2B). The data shown in Fig. 2b indicate that GBM cells partially lost their invasive ability to penetrate through extracellular matrix (ECM) as numbers of cells successfully reached the membrane part of the chamber shown in the chart significantly decreased in PP242-treated groups. On the other hand, treatment with rapamycin did not exhibit effects on invasion. Similarly, effects of U0126 were significantly less than that observed with PP242. These invasion assay results suggest that inhibition of mTORC2 has significant effect on GBM invasion.

### Purification of mTORC2 reveals association of Filamin A with mTORC2 in glioblastoma cells

**Fig. 3:**
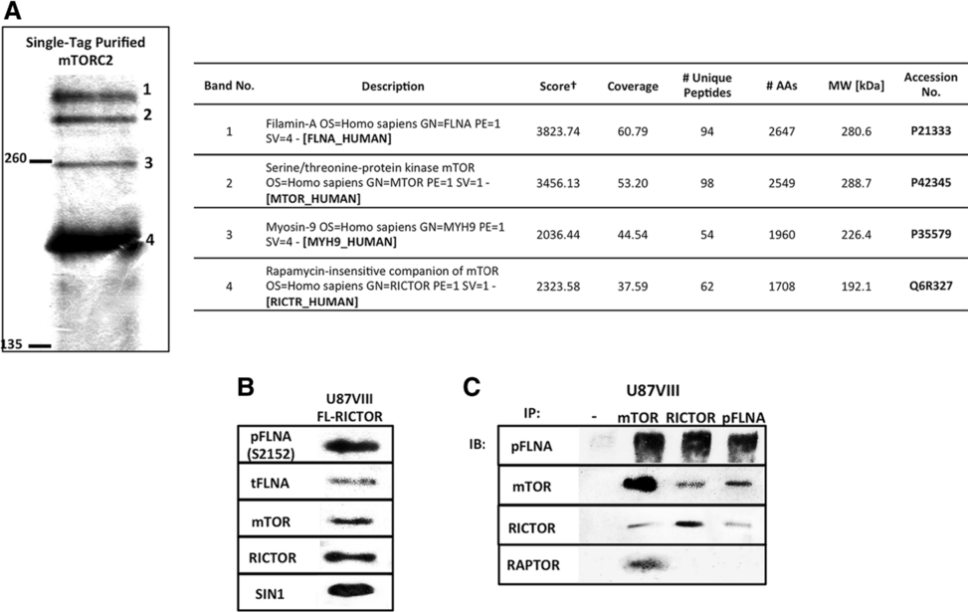
Purification, identification, and characterization of mTORC2 and its binding partners. **a** Silver-stained high molecular weight mTORC2 components purified from U87vIII cells stably expressing FLAG-RICTOR are shown. Four large proteins (numbered as 1-4) were analyzed by mass spectrometry after being run on 7 % SDS-PAGE gel, excised, then digested by Trypsin. Mass spectrometry results are summarized in the table on the right. Filamin A (FLNA) and Myosin-9 (MYH9) represent unknown bands 1 and 3, respectively. †Mascot protein score; a protein with score value >21 with >2 unique peptides is considered significant (P <0.05). **b** Immunoblots of purified proteins from U87vIII cells showing main components of mTORC2 (mTOR, RICTOR, SIN1) including phosphorylated and total FLNA. **c** Immunoprecipitation of U87vIII cell lysate by protein G Dynabeads coupled with antibodies against main components of mTOR complex 1 and 2. Phosphorylated FLNA was co-immunoprecipitated with anti-mTOR and anti-RICTOR coated beads while these two components of mTORC2 were pulled down with anti-pFLNA coated beads. RAPTOR, an mTORC1-specific component, can be co-immunoprecipitated with anti-mTOR coated beads but not with anti-pFLNA beads

The above results imply that mTORC2 plays critical roles in actin cytoskeleton and motility/invasion of GBM cells. To gain insight into how mTORC2 influences these events, we purified and characterized mTORC2 complexes in U87vIII cells. We stably expressed FLAG-RICTOR in GBM cell line U87vIII. The mTORC2 complex was prepared from U87vIII cells by carrying out affinity purification using FLAG-M2 magnetic beads. The purified mTORC2 was characterized by SDS polyacrylamide gel electrophoresis. In particular, we looked for mTORC2 associated proteins in a molecular weight range larger than 135 kDa. As shown in Fig. 3a, in addition to RICTOR, we detected three bands. Mass spectrometry was used to identify the proteins in the gel bands. Of the four bands, we found that the bands number 2 and 4 in Fig. 3a are mTOR and RICTOR, respectively. Proteins in bands 1 and 3 were found to be Filamin A (FLNA) and Myosin-9 (MYH9), respectively (Fig. 3a, right panel). FLNA is a large cytoplasmic non-muscle actin binding protein functioning as a signaling scaffold of cytoskeletal network [36]. MYH9 is known as non-muscle myosin heavy chain IIA, involved in several processes related to cellular motility such as cytoskeleton reorganization, focal contacts formation, and cell migration [37]. It is interesting that both proteins are associated with actin cytoskeleton dynamics. Because FLNA is directly involved with actin cytoskeleton, we focused our attention on FLNA.

The identity of components of mTORC2 pulled down by anti-FLAG antibody was further confirmed by Western blot analysis (Fig. 3b). As shown, in addition to mTOR, we identified SIN1 and FLNA proteins. FLNA is a phosphorylated protein modified most notably at Ser2152 [38]. Therefore, we used anti-pFLNA (Ser2152) antibody to see whether FLNA associated with mTORC2 is phosphorylated. As shown in Fig. 3b, we detected phospho-FLNA band. When mTORC2 is precipitated by the use of anti-mTOR antibody, we detected bands for RICTOR and phospho-FLNA, as well as RAPTOR (Fig. 3c). A similar experiment using anti-RICTOR antibody identified mTOR and phospho-FLNA. On the other hand, immunoprecipitation using anti-pFLNA (Ser2152) antibody precipitated mTOR and RICTOR but not RAPTOR. These results suggest that FLNA is specifically associated with mTORC2 and not mTORC1.

### RICTOR interacts with Filamin A

**Fig. 4:**
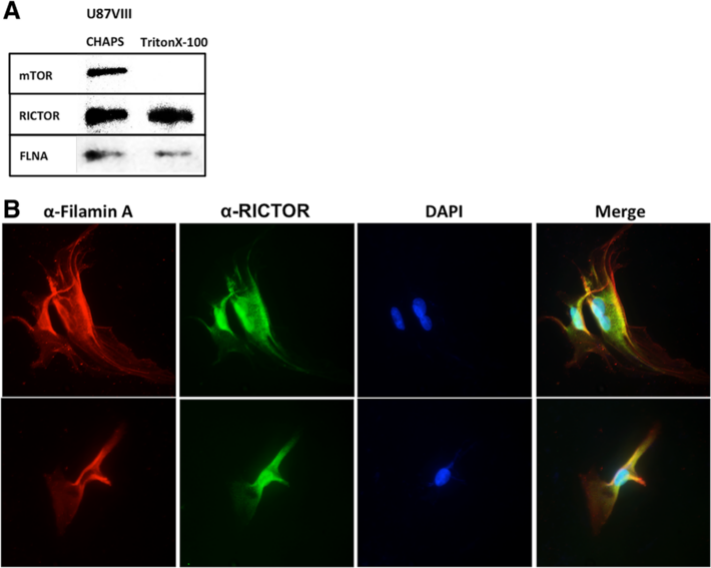
RICTOR binds FLNA. **a** FLAG-RICTOR is pulled down after lysing U87vIII cells with CHAPS or Triton X-100 as a detergent in lysis buffer. An immunoblot shows that RICTOR is associated with mTOR and FLNA in samples from CHAPS-treated extracts but only with FLNA in Triton X-100-treated extracts **b** Immunofluoresence labeling of two individual U87vIII cells grown in normal condition. Majority of FLNA and RICTOR colocalize along cell membrane and cytoplasm. Anti-FLNA antibody and Anti-RICTOR antibody were used to stain FLNA and RICTOR, respectively (Exposure time: FLNA 334 ms; RICTOR 540 ms; DAPI 41 ms). However, only RICTOR is found in nuclei of the cells. Overall results indicate that FLNA is an mTORC2 associated protein which is a binding partner of RICTOR

Because mTORC2 has multiple proteins associated, we asked which protein interacts with FLNA. To investigate this point, we lysed the cells with Triton X-100, a detergent that dissociates mTOR from its partners RAPTOR or RICTOR [39, 40]. We confirmed that the preparation of anti-FLAG pulldown obtained in the presence of 1 % Triton X-100 was devoid of mTOR protein (Fig. 4a). However, FLNA was still associated with the FLAG-RICTOR pulldown. In contrast, when CHAPS was used, RICTOR interacted with mTOR as well as with FLNA. These results suggest that FLNA is associated with RICTOR but not with mTOR.

Further support for the interaction between FLNA and RICTOR was obtained by the demonstration that these proteins colocalize in U87vIII cells. Fig. 4b shows immunofluorescence staining of two different GBM cells using anti-FLNA or anti-RICTOR antibody. DAPI staining was carried out to stain the nucleus. We found that most of FLNA and RICTOR colocalize with each other, especially the ones residing along the cell membrane (see yellow staining in the merged figure in Fig. 4b). In addition, mTOR also colocalizes with FLNA as shown in Additional file 3: Figure S3. These results show that RICTOR, mTOR and FLNA are physically located close by.

### Filamin A is phosphorylated by mTORC2 *in vitro*

**Fig. 5:**
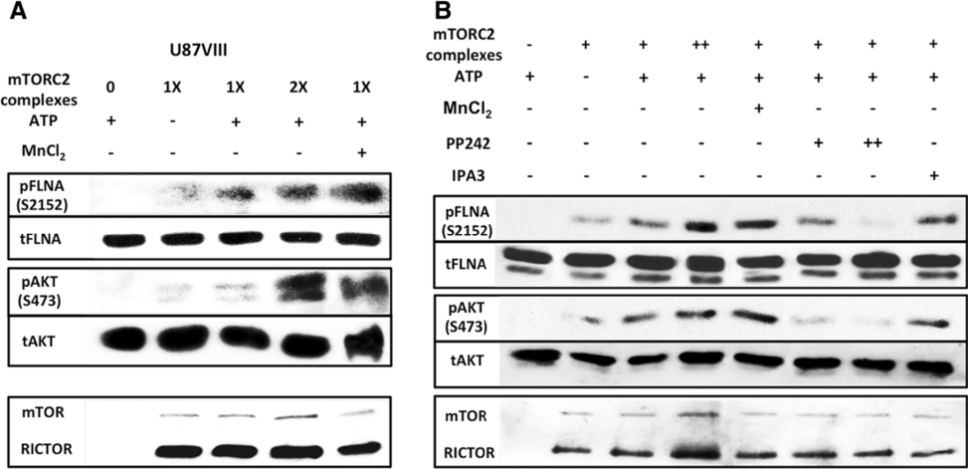
Purified mTORC2 phosphorylates FLNA at Ser2152 *in vitro*. **a** *In vitro* kinase assay of mTORC2 purified from U87vIII cells with FLNA and AKT as substrates. Level of phosphorylated FLNA (Ser2152) and phosphorylated AKT (Ser473) were increased in the presence of mTORC2 and ATP. Negative control groups contain either no purified complex or no ATP. Activity of mTORC2 is maximal when Mn^2+^ is used instead of Mg^2+^. **b** *In vitro* kinase assay of mTORC2 purified from U87vIII cells with FLNA as a substrate. Two concentrations of PP242 (+ = 1.25 μM; ++ = 5.0 μM) and one concentration of IPA3 (+ = 40 μM) were added into three samples to inhibit the kinase activity of mTORC2. Levels of pFLNA and pAKT when treated with PP242 are decreased compared to ones without PP242

In the previous experiments, we showed that FLNA associated with mTORC2 is phosphorylated (Fig. 3b and c). This led us to examine whether FLNA serves as a substrate for mTORC2 kinase activity. mTORC2 was purified from U87vIII cells and phosphorylation of FLNA at its regulatory residue Ser2152 was examined using FLNA C-terminal fragment as a substrate. As shown in Fig. 5a, dose-dependent phosphorylation of FLNA was detected. In this experiment, phosphorylation of AKT residue Ser473 was used as a positive control. Similar results were obtained with mTORC2 purified from HEK293T cells (Additional file 4: Figure S4C). Additionally, we confirmed that phosphorylation of FLNA substrate was catalyzed specifically by mTOR kinase activity, not by other kinases potentially contaminating the mTORC2 preparation. This was performed by carrying out an *in vitro* kinase assay of which two samples included PP242 at 1.25 μM and 5.0 μM. The results shown in Fig. 5b demonstrate that PP242 promotes a dose-dependent, significant inhibition of FLNA phosphorylation compared to those without the inhibitor. In contrast, the level of phosphorylated FLNA is not reduced in the sample containing PAK1 inhibitor.

### Filamin A phosphorylation is regulated by mTORC2 in glioblastoma cells

**Fig. 6:**
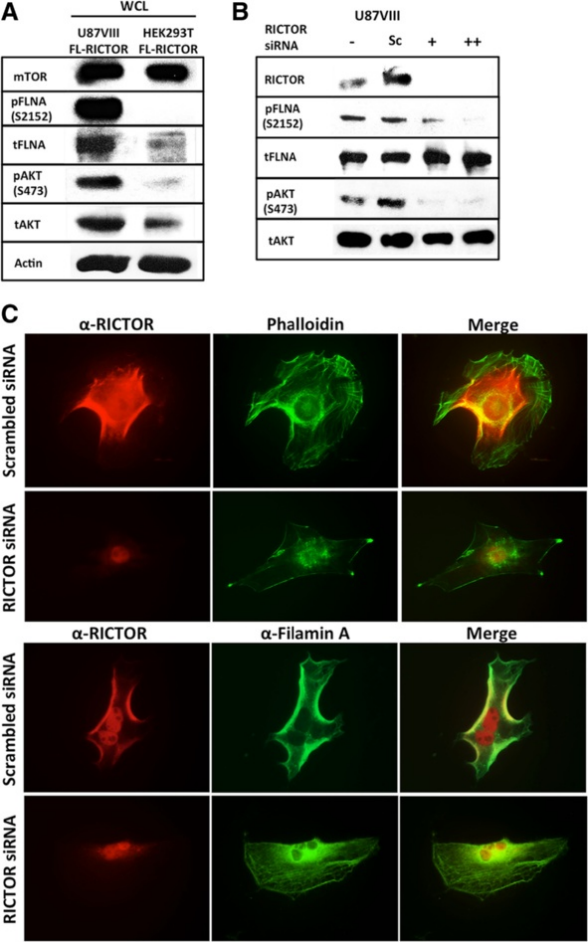
mTORC2 regulates FLNA phosphorylation at Ser2152. **a** An immunoblot showing high amounts of phosphorylated FLNA (pFLNA) and phosphorylated AKT (pAKT), a known mTORC2 substrate, in U87vIII cell lysate. In contrast, the amount of mTOR is similar in both U87vIII and HEK293T cells. **b** Level of FLNA phosphorylation (Ser2152) in U87vIII cells treated with two different concentrations of RICTOR siRNA decreases correlating to mTORC2 inhibition as detected by pAKT at Ser473. (Sc: scrambled siRNA, + = 4 μL, ++ = 8 μL siRNA). **c** Immunofluorescence staining of actin filaments, FLNA and RICTOR (Exposure time: RICTOR 318 ms; actin 580 ms; FLNA 123 ms). Results obtained from RICTOR knockdown experiment (48-h incubation with RICTOR siRNA) as described in Methods were used to observe effects of mTORC2 loss on arrangement of actin cytoskeleton and FLNA localization

We noticed that the levels of total and phosphorylated FLNA are significantly higher in GBM cells compared with HEK293T cells. This is shown in Fig. 6a. The basal level of FLNA in U87vIII is relatively higher than in HEK293T cells, while the amount of mTOR kinase is similar in both cell lines. More strikingly, we observed a dramatically higher level of phospho-FLNA (Ser2152) in U87vIII compared with HEK293T cells. The total amount of AKT is slightly higher in U87vIII cells and the amount of phospho-AKT is significantly higher in these cells. Thus, GBM cells exhibit dramatically increased levels of phosphorylated FLNA and AKT. Previous studies showed that the level of FLNA in more metastatic cancer cells is higher than in lower grade cancer cells of the same type [41, 42], suggesting the significance of FLNA in cancer progression and metastasis.

To investigate whether mTORC2 is responsible for FLNA phosphorylation at residue Ser2152 *in vivo*, we attempted to eliminate mTORC2 activity by depleting mTORC2 component, RICTOR, using siRNA. As shown in Fig. 6b, the level of RICTOR was significantly decreased by RICTOR siRNA but not by scrambled siRNA (Sc). The treatment with RICTOR siRNA resulted in the decrease of phosphorylated FLNA (Ser2152) in U87vIII cell lines while the total amount of FLNA was unchanged. The RICTOR siRNA treatment also decreased the level of phosphorylated AKT (Ser473). These results suggest that mTORC2 is a primary player in FLNA phosphorylation.

Moreover, inhibition of mTORC2 due to the depletion of RICTOR caused effects on actin filaments (Fig. 6c). Disordered and thin membrane-bound filaments were observed in a siRNA-treated group. FLNA proteins were also relocated in response to RICTOR knockdown. The shift of FLNA localization from the predominant membrane area to perinuclear/nuclei area is clear when comparing scrambled siRNA-treated group and RICTOR siRNA-treated group.

### PP242 inhibits Filamin A phosphorylation and causes dissociation of Filamin A from actin cytoskeleton

**Fig. 7:**
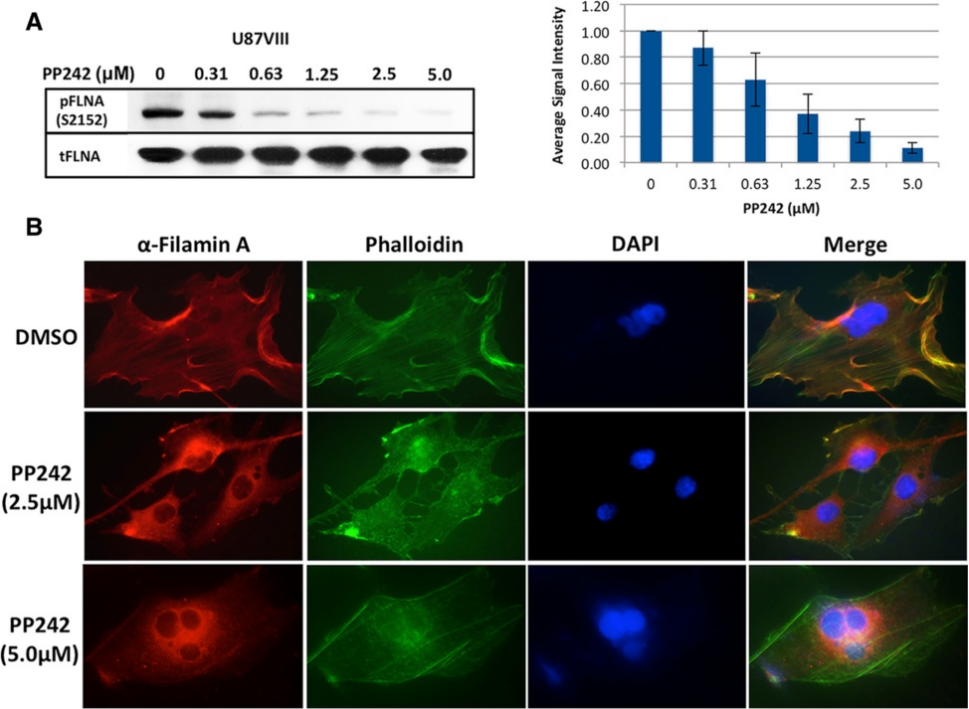
PP242 inhibits FLNA phosphorylation and causes dissociation of FLNA from actin cytoskeleton. **a** Levels of pFLNA (Ser2152) in U87vIII cells treated with different concentrations (0.31-5.0 μM) of PP242 were examined by carrying out Western analysis using anti-phospho-FLNA antibody. Average relative signal intensity of pFLNA adjusted by total FLNA amount of each condition is shown. Error bars indicate ± SD (*n* = 3). **b** Effects of PP242 on actin cytoskeleton and FLNA localization were examined by treating U87vIII cells with PP242 (2.5 μM or 5.0 μM) for 24 h before fixation. Immunofluorescence staining of actin filaments and FLNA was carried out as described in Methods (Exposure time: FLNA130 ms; actin 250 ms; DAPI 55 ms). Colocalization of Actin and FLNA is interrupted in samples treated with PP242. The results resemble effects of the treatment with siRNA against RICTOR

We treated U87vIII cells with varying concentrations of an mTOR ATP-competitive inhibitor PP242 that can inhibit mTORC2 kinase activity, and then the level of phospho-FLNA (Ser2152) was examined. As can be seen in Fig. 7a, the levels of phosphorylated FLNA decreased significantly in a dose-dependent manner. The amount of total FLNA, on the other hand, was unchanged by the treatment. PP242 also inhibited phosphorylation of FLNA (Ser2152) in HEK293T cells (Additional file 4: Figure S4D).

We found that FLNA colocalizes with actin cytoskeleton in GBM cells. As shown in Fig. 7b, the staining pattern of cells with anti-FLNA antibody looked similar to that observed with phalloidin staining (see the appearance of yellow fluorescence in the merged panel). After U87vIII cells were treated with 2.5 μM PP242 for 24 h, FLNA proteins and actin filaments were dissociated and were no longer colocalized, as revealed by the disappearance of yellow fluorescence in the merged panel. FLNA localization changes from plasma membrane to perinuclear area and into the nuclei. Stronger effects can be observed in cells treated with a higher concentration of PP242.

## Discussion

GBM has been depicted as one of the most invasive types of cancer [2, 43]. In this paper, we have shown that PP242, an ATP competitive inhibitor of mTOR, inhibits mTORC2 and exerts dramatic effects on actin cytoskeleton and focal adhesion in GBM cells. Moreover, PP242 causes inhibition of migration, as examined by scratch wound assay, while rapamycin or MEK inhibitor has only limited effect. In addition, PP242 inhibits invasion of GBM cells. By characterizing mTORC2 purified from GBM, we have found that Filamin A (FLNA) is an mTORC2 associated protein. This is confirmed by co-IP experiments. We have also shown that FLNA is phosphorylated by mTORC2 *in vitro* and *in vivo*. Inhibition of mTORC2 by RICTOR siRNA or PP242 treatment results in the inhibition of FLNA phosphorylation and dissociation of FLNA from actin cytoskeleton. Taken together, our results suggest that mTORC2/FLNA axis is critical for actin cytoskeleton, motility and invasion of GBM cells.

FLNA is ubiquitously expressed in non-muscle cells [38], and this 280 kDa actin binding protein serves as an essential organizer of cell structure and function by assisting in cell shape maintenance and organization of cytoskeletal networking, providing cell plasticity, and protecting cells from shearing stresses [44]. Because FLNA crosslinks cortical actin filaments to cell membrane, cell protrusions such as filopodia, lamellipodia, and pseudopodia can be initiated [36, 45]. FLNA also provides connection between various cytoskeletal proteins in cytoplasm to integrin localizing on the plasma membrane. In this manner, FLNA can anchor F-actin to various transmembrane proteins [46]. Getting released from FLNA, the integrin is activated and functional in propelling the cells [38, 47]. Thus, the mTORC2/FLNA axis plays important roles in cellular functions relating actin cytoskeleton, motility and invasion. In addition, both FLNA and mTORC2 could affect small GTPases known to control actin cytoskeleton (Rho), and chemotaxis and accumulation of F-actin (Rac/cdc42) [48].

By dissociating mTORC2 complex, we have shown that FLNA binds RICTOR but not mTOR. Since RICTOR is a specific component of mTORC2 (while RAPTOR is a specificity protein for mTORC1), this result explains specific effects of mTORC2 on actin cytoskeleton, motility and invasion. We further showed that RICTOR and FLNA are colocalized in GBM cells. Once associated with mTORC2, FLNA is phosphorylated by mTOR. This was shown first by demonstrating that mTORC2 purified from U87vIII cells could phosphorylate FLNA and this phosphorylation is inhibited by PP242. Second, knockdown of mTORC2 by applying RICTOR siRNA resulted in the inhibition of FLNA phosphorylation in U87vIII cells. These results suggest that FLNA is a new substrate of mTORC2. The specific site of phosphorylation, Ser2152, is located on FLNA’s repeat 20 that is involved with the migration and invasion properties of cancers [47]. In addition, this site is associated with the binding of the protein to C-terminal region of β-integrin [38], suggesting that multiple interactions occur at this specific location of FLNA, and pointing to the importance of Ser2152 in maintaining integrity of the protein and initiation of several cellular activities.

According to our findings, it appears that mTORC2-mediated phosphorylation of FLNA is a primary event in GBM migration and invasion, even though FLNA can be phosphorylated by multiple protein kinases including cyclic AMP (cAMP)-dependent protein kinase (PKA), p90 ribosomal kinase (RSK), PAK1, cyclin D1/Cdk4 and PKCα [49–52]. Our results shown in Additional file 1: Figure S1A demonstrate that MEK inhibitor (U0126), inhibiting upstream regulators of RSK, and PAK1 inhibitor (IPA3) did not significantly affect phosphorylation of FLNA compared to PP242. While FLNA is associated with mTORC2, we did not observe PKCα association with isolated complexes (Additional file 1: Figure S1B). Taken together, it appears that mTORC2 plays critical roles in the phosphorylation event of FLNA.

We found that FLNA is overexpressed and highly phosphorylated at Ser2152 in GBM cells. This presumably reflects high level of mTORC2 activation in these cells perhaps due to overexpression of RICTOR. We have shown that phosphorylation of FLNA by mTORC2 is important for cytoskeletal effects. Inhibition of FLNA phosphorylation by ATP-competitive mTOR inhibitor (PP242) leads to impaired FLNA-actin-plasma membrane linkages and disorganization of actin cytoskeleton in addition to the disruption of FLNA-actin colocalization. FLNA appeared to move inward to the perinuclear area after being inactivated by the inhibitor. Similarly, inhibiting mTORC2 by knocking down RICTOR results in the inhibition of FLNA phosphorylation and this causes dramatic change in actin cytoskeleton and FLNA localization. From the recent study, knockdown of FLNA impairs migration of neuronal cells, disrupts actin cytoskeleton and defects filopodia formation [53]. Therefore, both FLNA knockdown and inhibition of phosphorylation by PP242 affect actin cytoskeleton.

Our studies reinforce the idea that FLNA is a critical player in GBM. FLNA was firstly shown to be associated with P311 protein, and colocalize on the leading edges of glioma cells [54]. Highly expressed P311 is involved with invasive glioma migration by acting downstream of β1-integrin. Disruption of β1-integrin affects P311-mediated Rac1, Cdc42 activation, and finally inhibits cell migration [54]. Recent findings established that metastasis of GBM is related to the activity of calpain-2 enzyme, accounting for proteolysis of its substrates; Talin and FLNA through the maintenance of extracellular matrix metalloproteinases 2 (MMP2) [55, 56]. FLNA may be significant in migration, invasion and metastasis of other cancers also. From previous studies, FLNA appears to be overexpressed in several types of cancers, such as prostate cancer, breast cancer, lung cancer, colon cancer, melanoma, and neuroblastoma [47]. Involvement of FLNA in cancer metastasis was suggested in multiple other studies [57–59]. In addition, Zhang L *et al.* reported that overexpression of FLNA in *Tsc1*^null^ neurons promoted abnormal dendritic patterning which is a common shared feature of neurodevelopmental diseases [60].

Another protein that we found associated with purified mTORC2 is Myosin-9 (MYH9 or NMHC-IIA). MYH9 is a member of class II actin motors with ATPase activity that promote formation of lamellipodia, filopodia, and membrane ruffles [37, 61]. This machinery is critical for various developmental processes of the cells such as embryogenesis, organogenesis, and immune synapse formation [62]. Many kinases in several signaling pathways have been shown to regulate MYH9 [63]. Abnormalities of MYH9 and its associated proteins cause multiple types of diseases including cancers [62]. Also, MYH9 was reported to play important roles in breast cancer motility and glioma invasion [64, 65]. Further work is needed to investigate whether MYH9 is a component or substrate of mTORC2 and what role it plays in mTORC2 function.

In summary, our results highlight the importance of mTORC2-FLNA lineage in glioblastoma multiforme, and suggest that FLNA is associated with mTORC2 as a new downstream target. mTORC2 activates FLNA by phosphorylating its biological regulatory site Ser2152 which elicits actin cytoskeletal changes and further affects cell motility and invasiveness. Future work should uncover signaling events involving the mTORC2-FLNA axis in GBM.

## Conclusions

Taken together, our results provide the evidence to demonstrate that FLNA is a new downstream effector of mTORC2. Inhibition of mTORC2 resulted in the decreased level of phosphorylated FLNA, and led to defects in the organization of actin networks. Therefore, we propose that the new mTORC2-FLNA signaling pathway could be the key regulator in motility and invasion of glioblastoma cells.

## Methods

### Antibodies and other reagents

Anti-FLAG M2 magnetic beads, 3X FLAG peptide, Phalloidin-FITC, anti-Vinculin (VCL), and PP242 were obtained from Sigma-Aldrich. Anti-AU1 agarose beads were from BETHYL Lab. AU1 peptide (DTYRYI) was from COVANCE. Recombinant C-terminal fragment (amino acids 1730–2639) of Human FLNA purified from *E. coli* was obtained from Creative Biomart. Full-length inactive recombinant AKT1/PKB was from EMD Millipore. Anti-mTOR, Anti-phospho-AKT(Ser473), Anti-AKT, Anti-phospho-FLNA(Ser2152), Anti-phospho-S6(Ser235/236), Anti-S6, Anti-phospho-ERK (Thr202/Tyr204), and Anti-ERK were obtained from Cell Signaling. Anti-FLNA was obtained from EMD Millipore. Anti-RICTOR and anti-SIN1 were obtained from BETHYL Lab. Anti-phospho-CRKII and Anti-CRKII (Ser41) were obtained from Santa Cruz Biotechnology.

### Cell culture

U87 overexpressing EGFR vIII (U87vIII) and HEK293T cells were maintained in DMEM containing 10 % (vol/vol) FBS, 1 % (vol/vol) penicillin/streptomycin at 37 °C with 5 % (vol/vol) CO_2_. Stable U87vIII and HEK293T cells expressing FLAG-RICTOR protein were established using lentivirus containing pRK-5-myc-RICTOR plasmid (Addgene plasmid # 1860) [29] generated by the UCLA Vector Core Facility.

### Drug treatment

U87vIII cells were cultured in DMEM containing 10 % (vol/vol) FBS, 1 % (vol/vol) penicillin/streptomycin at 37 °C with 5 % (vol/vol) CO_2_ until they reached 80 % confluency. Cells were serum starved in serum-free media for 24 h prior to a 24-h treatment of different concentrations of rapamycin or PP242 in regular culturing media. We have shown that PP242 at the highest concentration (5 μM) did not inhibit ERK or PAK1 activity, confirming specific effects on mTORC2 prior to its usages in other experiments (Additional file 1: Figure S1A).

### Western blotting

Cultured cells were lysed with a lysis buffer containing 0.2 % Triton X-100 and protease inhibitor cocktail (EDTA-free PIC; Roche). The amount of total protein concentration in cleared lysate was determined by Bio-Rad protein assay. Equal protein extracts from various samples were separated by electrophoresis on SDS-PAGE gel, and then transferred to a nitrocellulose membrane (GE Healthcare). The membrane was blocked in Tris-buffered saline containing 0.05 % Tween20 and 5 % bovine serum albumin, then probed with primary antibodies followed by secondary antibodies (Horseradish peroxidase (HRP)-conjugated). The blot was incubated in Pierce ECL Western Blotting Substrate solution (Thermo Scientific). Protein bands from peroxidase activities to chemiluminescent substrates were developed and detected on films.

### Immunofluorescence

U87vIII cells were fixed with 4 % Paraformaldehyde, lysed with 0.2 % Triton X-100 buffer, blocked with 1 % BSA, then incubated in primary antibodies (anti-FLNA, anti-VCL, anti-RICTOR, anti-mTOR, FITC-phalloidin) overnight at 4 °C or 3 h at room temperature. Anti-mouse (Texas Red conjugate), and anti-rabbit (Alexa Flour 594 conjugate and Alexa Flour 488 conjugate) secondary antibodies from Life Technology were used. Nuclei of cells were stained by DAPI. FLNA, Actin filaments, RICTOR, and VCL, were observed using a fluorescence microscope.

### Wound healing migration assay

U87vIII cells were plated onto several 35 mm collagen-coated culture dishes (Sigma- Aldrich) until the surface of each dish was covered by a monolayer of cells. A single scratch per plate was made using a small pipette tip creating a 1 mm wide linear gap. Phase contrast images of all sample groups were taken at hour 0 before drug administration. Cells were treated with PP242 2.5 μM, rapamycin 100 nM, U0126 5 μM for 12 h, then images were acquired again.

### Transwell invasion assay

U87vIII cells were serum starved for 24 h prior to the experiment. One hundred thousand cells were treated with DMSO or drugs (rapamycin, PP242, or U0126) at different concentrations for 6 h in serum-free DMEM. Then, the media was changed to new 200 μl of serum-free media without drugs. Cells in drug-free media were transferred to an upper chamber of a 6.5 mm transwell insert with a 8.0 μm pore polycarbonate membrane (modified Boyden chamber) coated with 2-3 mg/ml basement matrix extract (BME) (Cultrex; Trevigen). In each lower chamber we added 500 μl of DMEM with 10 % FBS. The 24-well plates were incubated at 37 °C for 24 h. After that, DMEM and BME in the upper compartment were removed, and cells on the bottom of the membrane were washed in PBS. The membranes were then fixed in chilled methanol for 10 min following by crystal violet staining for 25 min. Cells were destained by acetic acid, and transferred to a 96-well plate. Absorbance representing relative cell numbers of each sample group was detected at 590 nm. Cell migration of each group was determined with the use of modified Boyden chamber without BME coating. Absorbance value from migrated cells of the control group (with DMSO) represented one hundred thousand cells. Absolute numbers of invaded cells under different conditions were calculated by comparing the ratio of absorbance values from invaded cells to migrated cells.

### Statistical analysis

Statistical analysis of cell invasion assay was performed using absolute numbers of invaded cells from sample groups. Statistically significant differences between two data sets were assessed with the two-tailed, unpaired Student’s *t*-test, with *P-*values of *P* < 0.05 sufficient to reject null hypothesis.

### Purification of mTORC2

U87vIII and HEK293T cells were infected with the FLAG-RICTOR lentivirus and cells stably expressing FLAG-RICTOR were obtained by puromycin selection. The drug-selected cell lines were cultured and grown, collected after washing with PBS, and stored at -80 °C for subsequent experiments. To purify mTORC2, cells were lysed in lysis buffer (50 mM HEPES, pH 7.4, 150 mM NaCl, 2 % CHAPS, 1X Complete EDTA-free protease inhibitor mixture (Roche Applied Science), and 1 mM Na_3_VO_4_). Cleared supernatant after centrifugation (16,000 × g for 10 min) was mixed with anti-FLAG M2 magnetic beads (Sigma-Aldrich) for affinity purification. The beads were collected, washed twice with wash buffer containing ATP (50 mM HEPES, pH 7.4, 150 mM NaCl, 2 mM DTT, 2 mM ATP, 0.1 % CHAPS), and washed three times with high salt wash buffer without ATP (50 mM HEPES, pH 7.4, 300 mM NaCl, 2 mM DTT, 0.1 % CHAPS). The bound proteins were eluted from magnetic beads using 3X FLAG peptide (Sigma-Aldrich) in 50 mM HEPES, pH 7.4, 500 mM NaCl, 0.4 % CHAPS. Eluted proteins were concentrated using Amicon Ultra 0.5-ml centrifugal filters NMWL 100 K (EMD Millipore, Billerica, MA). For the experiment that required dissociation of mTOR from RICTOR, 1 % Triton X-100 was substituted for 2 % CHAPS in the lysis buffer. When necessary, we included additional affinity purification to remove excess RICTOR by co-expressing AU1-mTOR and using AU1-agarose beads. This yielded mTORC2 complexes containing approximately equal amounts mTOR and RICTOR proteins. This method is called two-step purification. To perform this method, we first transfected HEK293T stably expressing FLAG-RICTOR with AU1-mTOR DNA for 48 h before cell collection. After FLAG-beads purification, mTORC2 was further subjected to another round of affinity purification using AU1 beads and eluted with AU1 peptide. Silver staining and Western blotting were performed to analyze the purified mTORC2.

### Mass spectrometry

#### In-gel trypsin digests and LC-MS/MS

The mTORC2 preparation was fractionated by SDS-PAGE and gel slices from the 200–300 kDa MW region were excised for MS analysis. Gel slices stained with Gelcode Blue stain (Thermo Scientific) were washed with a 1:1 mixture of 100 mM ammonium bicarbonate (ABC) and acetonitrile. The gel was further destained with 100 % acetonitrile and dried down by vacuum centrifugation. Proteins in the gel slices were reduced with 10 mM DTT at 60 °C for 1 h followed by alkylation with 50 mM iodoacetamide at 45 °C for 45 min. Extensive washing with 100 mM ammonium bicarbonate and acetonitrile was performed prior to overnight trypsin digestion with 15 ng sequencing grade trypsin (Promega). The resulting tryptic peptides were extracted from the gel slices with 30 μL of 50 % acetonitrile, 1 % TFA. Extraction was repeated a total of three times and the extracted peptides were pooled prior to drying down by vacuum centrifugation. Samples were reconstituted in 15 μL 1 % formic acid in water for MS analysis.

Tryptic peptides extracted from the gel bands were analyzed by LC-MS/MS using an EASY-nLA 1000 HPLC (Thermo Scientific) coupled to a Q-Exactive Orbitrap mass spectrometer (Thermo Scientific) equipped with an EASY-Spray nano-ESI source. 5 μL of each tryptic digest sample were injected onto a 75 μm X 15 cm, 3 μ, 100 Å PepMap C18 reversed-phase analytical LC column and separated with a linear gradient of 100 % solvent A (0.1 % formic acid in water) to 30 % solvent B (0.1 % formic acid in acetonitrile) over 20 min at a constant flow rate of 300 nL/min. Samples were analyzed using a Top 10 data-dependent acquisition method with 70,000 and 17,500 resolution at *m/z* 200 for MS1 and MS2 analysis respectively. Raw data files were processed in Proteome Discoverer (version 1.4, Thermo Scientific) using MASCOT (version 2.4.1; Matrix Science, London, UK) database searching to identify proteins entrapped in the 200–300 kDa gel slices. Tryptic peptides with up to 2 missed cleavages were searched against the SwissProt human database (2013) with the following settings: precursor and product ion mass tolerances of 10 ppm and 0.8 Da respectively, dynamic modification for oxidation (M), static modification for carbamidomethyl (C).

### Co-Immunoprecipitation

U87vIII cells were lysed with 2 % CHAPS lysis buffer and centrifuged at 13200 RPM for 10 min. Cleared supernatant was divided into groups to be incubated with primary antibodies against mTOR, phospho-FLNA (Ser2152), RICTOR and RAPTOR overnight at 4 °C. Control groups have no antibody. Then, Protein G superparamagnetic beads (Dynabeads; Life Technologies) were added to each sample group and were rotated for 30-45 min at 4 °C. Immunoprecipitated and co-immunoprecipitated proteins were detected using Western blotting analysis.

### siRNA treatment

On-TARGET plus Smartpool Human RICTOR siRNA was purchased from Thermo (Cat# 016984-00). U87vIII cells were transfected using Lipofectamine RNAi max (Life Technologies) and RICTOR siRNA or siGENOME-non-targeting #1 (D-001206-13-05) for 24 h before changing the media. All samples were collected 48 h after siRNA treatment. Western blotting was performed to show effects of RICTOR knockdown on FLNA phosphorylation. For immunofluorescence experiment, siRNA transfected cells were transferred to 4-well chamber slides 24 h after siRNA treatment and incubated for another 24 h. Cells were then fixed, treated with anti-FLNA, anti-RICTOR, phalloidin, followed by secondary antibodies, and were observed under the fluorescent microscope.

### *In vitro* kinase assay

Kinase activities of mTORC2 from U87vIII cells expressing FLAG-RICTOR were examined in various conditions using affinity purified mTORC2 as described above. Purified FLNA protein (C-terminal fragment amino acid 1730–2639; Creative Biomart) from *E. coli* and purified AKT protein (full-length; EMD Millipore) were used as substrates for the kinase assay. In each reaction, purified mTORC2 and its substrate (FLNA or AKT) were incubated in a buffer containing 20 mM Tris-HCl (pH 7.5), 10 mM MgCl_2_, 0.2 mM ATP for 25 min. Negative control reactions contain either no ATP or no mTORC2. MnCl_2_ was added to the positive control reaction. PP242 at two concentrations, 1.25 μM and 5 μM, and 40 μM of IPA3 were added to the reactions to observe mTOR kinase inhibition. Phosphorylation of FLNA (Ser2152) and AKT (Ser473) were detected by Western blot analysis.

## Additional files

Additional file 1: Figure S1.
**A**. PP242 inhibits mTORC2 while PAK1 and MEK inhibitors do not. Cells were treated with inhibitors (PP242, U0126, or IPA3) for 24 h and levels of phosphorylated FLNA, AKT, ERK, and CRKII were examined by Western blotting analysis. **B**. RICTOR and mTOR can be detected in either purified mTORC2 (Complex) or whole cell lysate (WCL) from U87vIII cells while PKCα was found only in WCL. Additional file 2: Figure S2.
**A**. The treatment of PP242 at high concentration (5 μM) does not affect U87vIII cell density. Phase contrast pictures were taken at 24 h after the treatment. **B**. Invaded U87vIII cells from different conditions, stained by crystal violet, on the membranes of modified Boyden chambers are shown. Pictures were taken after a 24-h invasion assay. (Ctrl = DMSO, P2.5 = PP242 2.5 μM, P5.0 = PP242 5.0 μM, R = rapamycin 100 nM, U = U0126 10.0 μM). Additional file 3: Figure S3. mTOR colocalizes with FLNA. Immunofluorescence staining of U87vIII cells grown in normal condition shows localization of mTOR and FLNA (Exposure time: FLNA 180 ms; mTOR 525 ms; DAPI 42 ms). Both proteins are found colocalized along the cell membrane. Additional file 4: Figure S4. Results obtained from HEK293T cells **A**. Silver-stained mTORC2 components (high molecular weight) purified from HEK293T cells stably expressing FLAG-RICTOR are shown. Four large proteins are numbered and labeled. **B**. Immunoblots of purified proteins from HEK293T cells showing main components of mTORC2 (mTOR, RICTOR, SIN1) including phosphorylated and total FLNA. **C**. *In vitro* kinase assay of mTORC2 purified from HEK293T cells with FLNA and AKT as substrates. Level of phosphorylated FLNA (Ser2152) and phosphorylated AKT (Ser473) were increased in the presence of mTORC2 and ATP. **D**. Western blot shows levels of pFLNA (Ser2152) in HEK293T cells treated with different concentrations (0.31-5.0 μM) of PP242.

## Abbreviations

mTOR: Mechanistic target of rapamycin
RICTOR: Rapamycin-insensitive companion of mTOR
FLNA: Filamin A
MYH9: Myosin-9
AKT: RAC-alpha serine threonine protein kinase or Protein kinase B
GBM: Glioblastoma multiforme
U87vIII: U87 cell line overexpressing epidermal growth factor receptor variant III
siRNA: short interfering RNA
ECM: Extracellular matrix

## References

[1] Louis DN, Ohgaki H, Wiestler OD, Cavenee WK, Burger PC, Jouvet A (2007). The 2007 WHO classification of tumours of the central nervous system. Acta Neuropathol.

[2] DeAngeles LM (2001). Brain Tumors. N Engl J Med.

[3] Hegi ME, Rajakannu P, Weller M (2012). Epidermal growth factor receptor: a re-emerging target in glioblastoma. Curr Opin Neurol.

[4] Akhavan D, Cloughesy TF, Mischel PS (2010). mTOR signaling in glioblastoma: lessons learned from bench to bedside. Neuro Oncol.

[5] Tanaka K, Babic I, Nathanson D, Akhavan D, Guo D, Gini B (2011). Oncogenic EGFR signaling activates an mTORC2-NF-κB pathway that promotes chemotherapy resistance. Cancer Discov.

[6] Huang PH, Miraldi ER, Xu AM, Kundukulam VA, Del Rosario AM, Flynn RA (2010). Phosphotyrosine signaling analysis of site-specific mutations on EGFRvIII identifies determinants governing glioblastoma cell growth. Mol Biosyst.

[7] Katanasaka Y, Kodera Y, Kitamura Y, Morimoto T, Tamura T, Koizumi F (2013). Epidermal growth factor receptor variant type III markedly accelerates angiogenesis and tumor growth via inducing c-myc mediated angiopoietin-like 4 expression in malignant glioma. Mol Cancer.

[8] Li L, Dutra A, Pak E, Labrie JE, Gerstein RM, Pandolfi PP (2009). EGFRvIII expression and PTEN loss synergistically induce chromosomal instability and glial tumors. Neuro Oncol.

[9] Vogt PK, Hart JR (2009). Akt demoted in glioblastoma. Sci Signal.

[10] Jhanwar-Uniyal M, Jeevan D, Neil J, Shannon C, Albert L, Murali R (2013). Deconstructing mTOR complexes in regulation of Glioblastoma Multiforme and its stem cells. Adv Biol Regul.

[11] Sabatini DM (2006). mTOR and cancer: insights into a complex relationship. Nat Rev Cancer.

[12] Adler EM (2011). 2010: Signaling Breakthroughs of the Year. Sci Signal.

[13] Angliker N, Rüegg MA (2013). In vivo evidence for mTORC2-mediated actin cytoskeleton rearrangement in neurons. Bioarchitecture.

[14] Thomanetz V, Angliker N, Cloëtta D, Lustenberger RM, Schweighauser M, Oliveri F (2013). Ablation of the mTORC2 component rictor in brain or Purkinje cells affects size and neuron morphology. J Cell Biol.

[15] Masri J, Bernath A, Martin J, Jo OD, Vartanian R, Funk A (2007). mTORC2 activity is elevated in gliomas and promotes growth and cell motility via overexpression of rictor. Cancer Res.

[16] Gulati N, Karsy M, Albert L, Murali R, Jhanwar-Uniyal M (2009). Involvement of mTORC1 and mTORC2 in regulation of glioblastoma multiforme growth and motility. Int J Oncol.

[17] Masui K, Tanaka K, Akhavan D, Babic I, Gini B, Matsutani T (2013). mTOR complex 2 controls glycolytic metabolism in glioblastoma through FoxO acetylation and upregulation of c-Myc. Cell Metab.

[18] Masui K, Cavenee WK, Mischel PS (2014). mTORC2 in the center of cancer metabolic reprogramming. Trends Endocrinol Metab.

[19] Schonbrun M, Kolesnikov M, Kupiec M, Weisman R (2013). TORC2 is required to maintain genome stability during S phase in fission yeast. J Biol Chem.

[20] Shimada K, Filipuzzi I, Stahl M, Helliwell SB, Studer C, Hoepfner D (2013). TORC2 signaling pathway guarantees genome stability in the face of DNA strand breaks. Mol Cell.

[21] Weisman R, Cohen A, Gasser SM (2014). TORC 2 — a new player in genome stability. EMBO Mol Med.

[22] Selvarajah J, Elia A, Carroll VA, Moumen A (2014). DNA damage-induced S and G2 / M cell cycle arrest requires mTORC2-dependent regulation of Chk1. Oncotarget.

[23] Zoncu R, Efeyan A, Sabatini DM (2011). mTOR: from growth signal integration to cancer, diabetes and ageing. Nat Rev Mol Cell Biol.

[24] Abraham RT (2004). PI 3-kinase related kinases : “big” players in stress-induced signaling pathways. DNA Repair (Amst).

[25] Sarbassov DD, Guertin DA, Ali SM, Sabatini DM (2005). Phosphorylation and regulation of Akt/PKB by the rictor-mTOR complex. Science.

[26] Sauer E, Imseng S, Maier T, Hall MN (2013). Conserved sequence motifs and the structure of the mTOR kinase domain. Biochem Soc Trans.

[27] Laplante M, Sabatini DM (2013). Regulation of mTORC1 and its impact on gene expression at a glance. J Cell Sci.

[28] Jacinto E, Loewith R, Schmidt A, Lin S, Rüegg MA, Hall A (2004). Mammalian TOR complex 2 controls the actin cytoskeleton and is rapamycin insensitive. Nat Cell Biol.

[29] Sarbassov DD, Ali SM, Kim D-H, Guertin DA, Latek RR, Erdjument-Bromage H (2004). Rictor, a novel binding partner of mTOR, defines a rapamycin-insensitive and raptor-independent pathway that regulates the cytoskeleton. Curr Biol.

[30] Jacinto E, Facchinetti V, Liu D, Soto N, Wei S, Jung SY (2006). SIN1/MIP1 maintains rictor-mTOR complex integrity and regulates Akt phosphorylation and substrate specificity. Cell.

[31] Ikenoue T, Inoki K, Yang Q, Zhou X, Guan K-L (2008). Essential function of TORC2 in PKC and Akt turn motif phosphorylation, maturation and signalling. EMBO J.

[32] Huang W, Zhu PJ, Zhang S, Zhou H, Stoica L, Galiano M (2013). mTORC2 controls actin polymerization required for consolidation of long-term memory. Nat Neurosci.

[33] Goncharova E (2014). a., James ML, Kudryashova T V., Goncharov D a., Krymskaya VP. Tumor Suppressors TSC1 and TSC2 Differentially Modulate Actin Cytoskeleton and Motility of Mouse Embryonic Fibroblasts. PLoS One.

[34] Apsel B, Blair JA, Gonzalez B, Nazif TM, Feldman ME, Aizenstein B (2008). Targeted polypharmacology: discovery of dual inhibitors of tyrosine and phosphoinositide kinases. Nat Chem Biol.

[35] Guo D, Prins RM, Dang J, Kuga D, Iwanami A, Soto H (2009). EGFR signaling through an Akt-SREBP-1-dependent, rapamycin-resistant pathway sensitizes glioblastomas to antilipogenic therapy. Sci Signal.

[36] Feng Y, Walsh CA (2004). The many faces of filamin : A versatile molecular scaffold for cell motility and signalling. Nat Cell Biol.

[37] Betapudi V (2010). Myosin II, motor proteins with different functions determine the fate of lamellipodia extension during cell spreading. PLoS One.

[38] Nakamura F, Stossel TP, Hartwig JH (2011). The filamins: Organizers of cell structure and function. Cell Adh Migr.

[39] Pearce LR, Huang X, Boudeau J, Pawłowski R, Wullschleger S, Deak M (2007). Identification of Protor as a novel Rictor-binding component of mTOR complex-2. Biochem J.

[40] Yip CK, Murata K, Walz T, Sabatini DM, Kang SA (2010). Structure of the Human mTOR Complex I and Its Implications for Rapamycin Inhibition. Mol Cell.

[41] Ai J, Huang H, Lv X, Tang Z, Chen T, Duan W (2011). FLNA and PGK1 are Two Potential Markers for Progression in Hepatocellular Carcinoma. Cell Physiol Biochem.

[42] Tian HM, Liu XH, Han W, Zhao LL, Yuan B, Yuan CJ (2013). Differential expression of filamin A and its clinical significance in breast cancer. Oncol Lett.

[43] Ohgaki H, Kleihues P (2007). Genetic pathways to primary and secondary glioblastoma. Am J Pathol.

[44] Yue J, Huhn S, Shen Z (2013). Complex roles of filamin-A mediated cytoskeleton network in cancer progression. Cell Biosci.

[45] MacPherson M, Fagerholm SC (2010). Filamin and filamin-binding proteins in integrin-regulation and adhesion. Focus on: “FilaminA is required for vimentin-mediated cell adhesion and spreading”. Am J Physiol Cell Physiol.

[46] Stossel TP, Condeelis J, Cooley L, Hartwig JH, Schleicher M, Shapiro SS (2001). Filamins as integrators of cell mechanics and signalling. Nat Rev Mol Cell Biol.

[47] Savoy RM, Ghosh PM (2013). The dual role of filamin a in cancer: can’t live with (too much of) it, can’t live without it. Endocr Relat Cancer.

[48] He Y, Li D, Cook SL, Yoon M-S, Kapoor A, Rao CV (2013). Mammalian target of rapamycin and Rictor control neutrophil chemotaxis by regulating Rac/Cdc42 activity and the actin cytoskeleton. Mol Biol Cell.

[49] García E, Stracher A, Jay D (2006). Calcineurin dephosphorylates the C-terminal region of filamin in an important regulatory site: a possible mechanism for filamin mobilization and cell signaling. Arch Biochem Biophys.

[50] Woo MS, Ohta Y, Rabinovitz I, Stossel P, Blenis J, Stossel TP (2004). Ribosomal S6 Kinase (RSK) regulates phosphorylation of filamin a on an important regulatory site. Mol Cell Biol..

[51] Vadlamudi RK, Li F, Adam L, Nguyen D, Ohta Y, Stossel TP (2002). Filamin is essential in actin cytoskeletal assembly mediated by p21-activated kinase 1. Nat Cell Biol.

[52] Tigges U, Koch B, Wissing J, Jockusch BM, Ziegler WH (2003). The F-actin cross-linking and focal adhesion protein filamin A is a ligand and in vivo substrate for protein kinase Cα. J Biol Chem.

[53] Zhang J, Neal J, Lian G, Hu J, Lu J, Sheen V (2013). Filamin A regulates neuronal migration through brefeldin A-inhibited guanine exchange factor 2-dependent Arf1 activation. J Neurosci.

[54] McDonough WS, Tran NL, Berens ME (2005). Regulation of glioma cell migration by seri ne-phosphorylated P311. Neoplasia.

[55] Jang HS, Lal S, Greenwood J (2010). a. Calpain 2 is required for glioblastoma cell invasion: regulation of matrix metalloproteinase 2. Neurochem Res.

[56] Lal S, La Du J, Tanguay RL, Greenwood JA (2012). Calpain 2 is required for the invasion of glioblastoma cells in the zebrafish brain microenvironment. J Neurosci Res.

[57] Sadanandam A, Lyssiotis CA, Homicsko K, Collisson EA, Gibb WJ, Wullschleger S (2013). A colorectal cancer classification system that associates cellular phenotype and responses to therapy. Nat Med.

[58] Zhang K, Zhu T, Gao D, Zhang Y, Zhao Q, Liu S (2014). Filamin A expression correlates with proliferation and invasive properties of human metastatic melanoma tumors: implications for survival in patients. J Cancer Res Clin Oncol.

[59] Alper O, Stetler-Stevenson WG, Harris LN, Leitner WW, Ozdemirli M, Hartmann D (2009). Novel anti-filamin-A antibody detects a secreted variant of filamin-A in plasma from patients with breast carcinoma and high-grade astrocytoma. Cancer Sci.

[60] Zhang L, Bartley CM, Gong X, Hsieh LS, Lin TV, Feliciano DM (2014). MEK-ERK1/2-Dependent FLNA Overexpression Promotes Abnormal Dendritic Patterning in Tuberous Sclerosis Independent of mTOR. Neuron.

[61] Sellers JR (2000). Myosins: a diverse superfamily. Biochim Biophys Acta - Mol Cell Res.

[62] Betapudi V (2014). Life without double-headed non-muscle myosin II motor proteins. Front Chem.

[63] Dulyaninova NG, Bresnick AR (2013). The heavy chain has its day: Regulation of myosin-II assembly. Bioarchitecture.

[64] Dulyaninova NG, House RP, Betapudi V, Bresnick AR (2007). Myosin-IIA heavy-chain phosphorylation regulates the motility of MDA-MB-231 carcinoma cells. Mol Biol Cell.

[65] Beadle C, Assanah MC, Monzo P, Vallee R, Rosenfeld SS, Canoll P (2008). The role of myosin II in glioma invasion of the brain. Mol Biol Cell.

