# Supplementary figures and images for: Significance of filamin A in mTORC2 function in glioblastoma
Source: Mol Cancer. 2015 Jul 2;14:127. doi: 10.1186/s12943-015-0396-z (PMC4489161; doi:10.1186/s12943-015-0396-z)

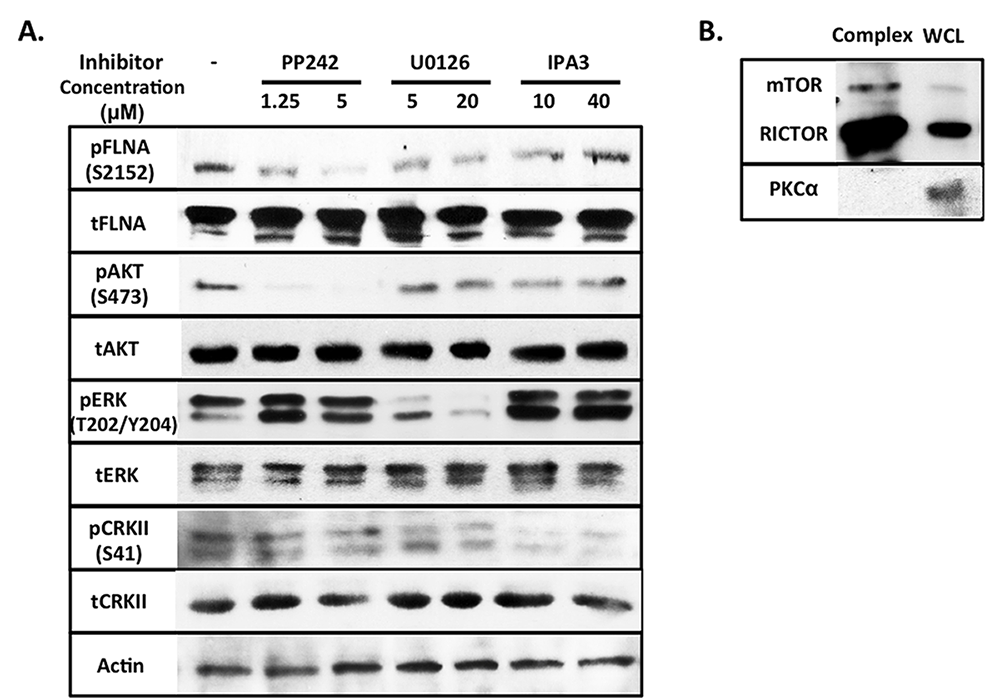

Supplement: Additional file 1: Figure S1. — A. PP242 inhibits mTORC2 while PAK1 and MEK inhibitors do not. Cells were treated with inhibitors (PP242, U0126, or IPA3) for 24 h and levels of phosphorylated FLNA, AKT, ERK, and CRKII were examined by Western blotting analysis. B. RICTOR and mTOR can be detected in either purified mTORC2 (Complex) or whole cell lysate (WCL) from U87vIII cells while PKCα was found only in WCL. [file 12943_2015_396_MOESM1_ESM.tif]

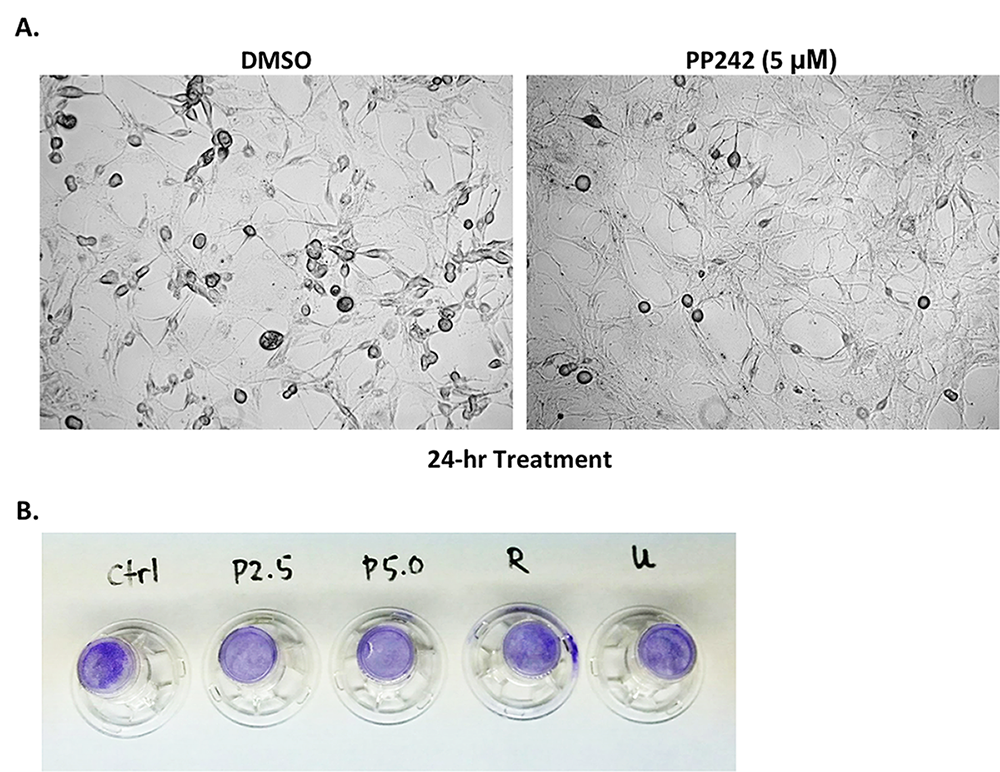

Supplement: Additional file 2: Figure S2. — A. The treatment of PP242 at high concentration (5 μM) does not affect U87vIII cell density. Phase contrast pictures were taken at 24 h after the treatment. B. Invaded U87vIII cells from different conditions, stained by crystal violet, on the membranes of modified Boyden chambers are shown. Pictures were taken after a 24-h invasion assay. (Ctrl = DMSO, P2.5 = PP242 2.5 μM, P5.0 = PP242 5.0 μM, R = rapamycin 100 nM, U = U0126 10.0 μM). [file 12943_2015_396_MOESM2_ESM.tif]

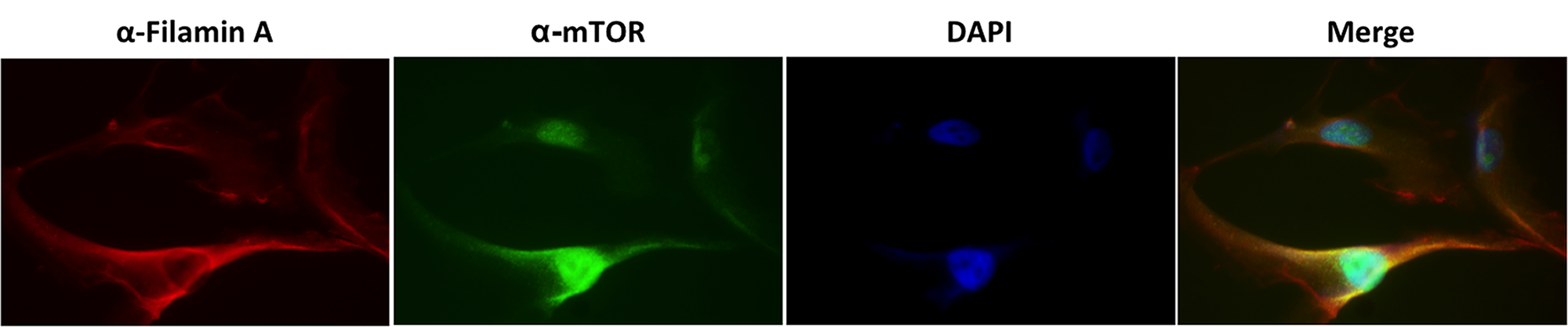

Supplement: Additional file 3: Figure S3. — mTOR colocalizes with FLNA. Immunofluorescence staining of U87vIII cells grown in normal condition shows localization of mTOR and FLNA (Exposure time: FLNA 180 ms; mTOR 525 ms; DAPI 42 ms). Both proteins are found colocalized along the cell membrane. [file 12943_2015_396_MOESM3_ESM.tif]

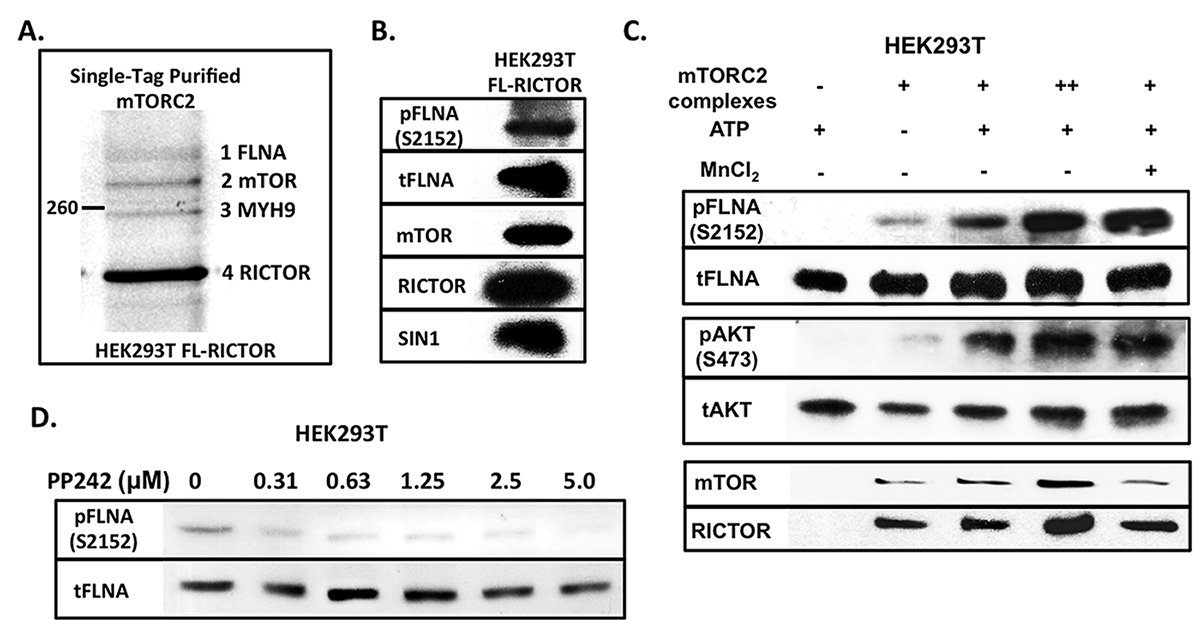

Supplement: Additional file 4: Figure S4. — Results obtained from HEK293T cells A. Silver-stained mTORC2 components (high molecular weight) purified from HEK293T cells stably expressing FLAG-RICTOR are shown. Four large proteins are numbered and labeled. B. Immunoblots of purified proteins from HEK293T cells showing main components of mTORC2 (mTOR, RICTOR, SIN1) including phosphorylated and total FLNA. C. In vitro kinase assay of mTORC2 purified from HEK293T cells with FLNA and AKT as substrates. Level of phosphorylated FLNA (Ser2152) and phosphorylated AKT (Ser473) were increased in the presence of mTORC2 and ATP. D. Western blot shows levels of pFLNA (Ser2152) in HEK293T cells treated with different concentrations (0.31-5.0 μM) of PP242. [file 12943_2015_396_MOESM4_ESM.tif]
